# Investigation of urine metabolome of BALB/c mouse infected with an avirulent strain of *Toxoplasma gondii*

**DOI:** 10.1186/s13071-022-05408-2

**Published:** 2022-07-29

**Authors:** Chun-Xue Zhou, Ling-Yu Li, Cui-Qin Huang, Xu-Dong Guo, Xu-Dian An, Fang-Fang Luo, Wei Cong

**Affiliations:** 1 Department of Pathogen Biology, School of Basic Medical Sciences, Cheeloo College of Medicine, Shandong University, Jinan, 250012 Shandong People’s Republic of China; 2 Engineering Research Center for the Prevention and Control of Animal Original Zoonosis, Fujian Province University & College of Life Science, Longyan University, Longyan, 364012 Fujian People’s Republic of China; 3 Marine College, Shandong University, Weihai, 264209 Shandong People’s Republic of China

**Keywords:** *Toxoplasma gondii*, Urine, Mass spectrometry, Metabolomics

## Abstract

**Background:**

The protozoan parasite *Toxoplasma gondii* is a major concern for human and animal health. Although the metabolic understanding of toxoplasmosis has increased in recent years, the analysis of metabolic alterations through noninvasive methodologies in biofluids remains limited.

**Methods:**

Here, we applied liquid chromatography-tandem mass spectrometry (LC-MS/MS)-based metabolomics and multivariate statistical analysis to analyze BALB/c mouse urine collected from acutely infected, chronically infected and control subjects.

**Results:**

In total, we identified 2065 and 1409 metabolites in the positive electrospray ionization (ESI +) mode and ESI − mode, respectively. Metabolomic patterns generated from principal component analysis (PCA) and partial least squares discriminant analysis (PLS-DA) score plots clearly separated *T. gondii*-infected from uninfected urine samples. Metabolites with altered levels in urine from *T. gondii*-infected mice revealed changes in pathways related to amino acid metabolism, fatty acid metabolism, and nicotinate and nicotinamide metabolism.

**Conclusions:**

This is the first study to our knowledge on urine metabolic profiling of BALB/c mouse with *T. gondii* infection. The urine metabolome of infected mouse is distinctive and has value in the understanding of *Toxoplasmosis* pathogenesis and improvement of treatment.

**Graphical Abstract:**

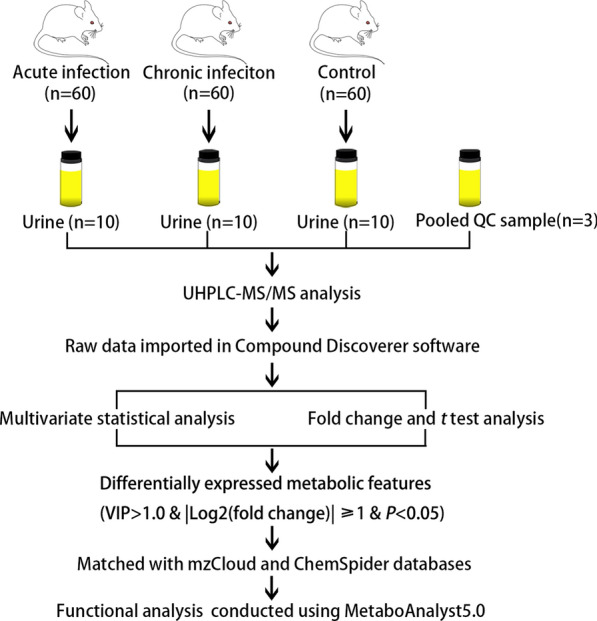

**Supplementary Information:**

The online version contains supplementary material available at 10.1186/s13071-022-05408-2.

## Background

*Toxoplasma gondii* is an obligate intracellular protozoan parasite belonging to the phylum Apicomplexa [1]. It infects almost all warm-blooded animals, including humans [2, 3]. Approximately one-third of the world’s human population carries this parasite [4]. Infections generally occur as a result of the inadvertent consumption of food or water contaminated by parasite oocysts shed in feline feces or the ingestion of tissue cysts in undercooked meat [5]. Infection is usually asymptomatic, whereas uncontrolled proliferation of the fast-replicating tachyzoites in immunocompromised individuals, including HIV patients and organ transplant recipients, can lead to severe consequences, such as encephalitis, retinochoroiditis and even death [6]. Additionally, this successful parasite can sometimes cross the placenta, resulting in dire health issues for the unborn baby or newborn infant. For instance, a miscarriage or stillbirth would happen if a primary infection happens during or just before pregnancy [7]. Unfortunately, there is no commercial vaccine to prevent infection or chemotherapeutic drug to eradicate this parasite from infected individuals [8, 9].

The diagnosis of a case of toxoplasmosis is established based on clinical manifestations, molecular techniques and serologic tests [10]. Amniotic fluid testing by polymerase chain reaction is useful in cases of possible mother-to-child transmission. Clinical diagnosis is more likely made by serologic methods, which mainly include indirect fluorescent antibody test and enzyme immunoassay. Although the detection of *T. gondii*-specific antibodies in patient serum is effective, an obvious shortcoming in *T. gondii* serodiagnosis is its inability to differentiate acute infection cases from long-term chronic ones [11]. So far, validated biomarkers that are used to reflect physiological or pathophysiological changes during toxoplasmosis have never been identified. Advances in techniques that detect and quantify multiple small chemical molecules in complex biological samples provide exciting new opportunities to identify small molecule biomarkers with high specificity and sensibility.

Metabolomics is the study of the chemical reactions found within cells and biological systems. It has been proved to be a useful tool in the identification of efficient drug targets or biomarkers for predicting the severity and progression of infectious diseases. Metabolome of tissues and body fluids should reflect biochemical dynamics during disease progression. As an obligate intracellular parasite, *T. gondii* relies on its hosts for resources, and metabolic flexibility is generally essential for *T. gondii* pathogenicity. Many studies have been performed to investigate metabolite variations during *T. gondii* infection using various approaches, including nuclear magnetic resonance (NMR) and liquid chromatography-mass spectrometry (LC-MS). For example, a metabolomic study based on LC-MS has revealed that *T. gondii* infection can induce extensive alterations in metabolism of the host cerebral cortex [12]. Another study on changes in the joint host-parasite metabolome over a time course of *Toxoplasma* infection revealed broad alterations to host metabolism by the parasite in both energetic and biosynthetic pathways [13]. Our previous metabolic study on mouse sera infected by *T. gondii *showed that pathways involved in host amino acid, lipid and energy signaling were remarkably disturbed [14]. Urine is viewed as a waste product with no biological role in the overall health of an individual. However, there is growing evidence that urine composition and properties reflect the generation of liquid by-products of ongoing metabolism and physiological conditions in the body. Nowadays, urine is being widely studied in clinical omics as it can be obtained noninvasively in large quantities and is stable in vitro. Urinary metabolomics has been studied in many infectious diseases, such as COVID-19 infection, Lyme disease and *Schistosoma* infection [15–17]. However, up to now, no such study has ever been performed on toxoplasmosis.

Therefore, to explore the urinary metabolome and get a better understanding of the pathophysiology of renal involvement in *T. gondii* infection, we used an untargeted liquid chromatography-tandem mass spectrometry (LC-MS/MS) platform, in light of its reliability, reproducibility and sensitivity, to profile a dynamic metabolomic landscape in host urine during toxoplasmosis progression. Comparative analysis of the data from infection and normal urine samples revealed changes in metabolic pathways such as amino acid and fatty acid metabolism. We hope data obtained in this study will benefit toxoplasmosis diagnosis and surveillance in a noninvasive and effective way.

## Methods

### Ethics statement

All animal experiments used in this study were approved by the Research Ethics Committee of Shandong University (ECSBMSSDU2020-2-027). The maintenance and care of experimental animals were carried out in strict accordance with the regulations of Good Animal Practice requirements of the Animal Ethics Procedures and Guidelines of the People’s Republic of China. All efforts were made to alleviate suffering and minimize the number of animals used in the study.

### Animals and parasites

Female mice (6–8 weeks old) were purchased from the Laboratory Animal Center of Shandong University, China. All mice were placed in an environment with appropriate temperature and ventilation, 12 h of light and 12 h of darkness. Mice were given sterilized food and water ad libitum. Mice were acclimated for 1 week before being used in the experiment.

*T. gondii* type II Prugniuad (Pru) strain was maintained in Kunming mice using oral inoculation with parasite cysts obtained from mice brain tissues 60 days post-infection (dpi). After anesthetizing animals, the infected brain tissues of the mice were removed and homogenized in a sterile tissue homogenizer. The cysts were counted and diluted to 100 cysts/ml in phosphate-buffered saline solution (PBS).

### Mouse infection and sample collection

Mouse infection and sample collection were performed as previously described [18]. Female BALB/c mice (6–8 weeks old, *n* = 180) were randomly divided into three groups (*n* = 60 per group): acutely infected group, chronically infected group and healthy control group. Mice in the infection group were administered intragastrically with ten *T. gondii* cysts suspended in 100 μl PBS. Control mice were gavaged with the same volume of PBS without parasites. Infected mice showed typical signs of acute toxoplasmosis 11 days after infection, and 24 h urine was collected from the acutely infected group. After 35 days of infection, all infected mice returned to normal but showed losses in body weight, and 24 h urine was collected from the chronically infected group. Each group contained ten biological replicates, with each biological replicate containing urine collected from six mice. To remove cellular debris, urine samples were centrifuged at 3000 g for 10 min at 4 °C. Supernatants were collected and frozen instantly in liquid nitrogen and stored at −80 °C until further use.

### Metabolite extraction

Urine samples were thawed on ice and mixed with four volumes of ice-cold methanol. Samples were vortexed and centrifuged at 14,000 g for 20 min. The supernatants were subsequently transferred to fresh precooled Eppendorf tubes and dried in a vacuum concentrator. Then, 100 µl of 60% ice-cold methanol was added, and the samples were vortexed. Finally, the supernatant was used for the following LC-MS/MS analysis. Meanwhile, quality control (QC) samples were used to evaluate the precision of the analytic system, which was produced by mixing and blending equal volumes of each sample.

### UHPLC-MS/MS analysis

UHPLC-MS/MS analyses were carried out using a Vanquish UHPLC system (ThermoFisher, Germany) coupled with an Orbitrap Q ExactiveTM HF mass spectrometer (Thermo Fisher, Germany) in the data-dependent acquisition mode. Samples were injected into a Hypesil Goldcolumn (100 × 2.1 mm, 1.9 µm) using a 16-min linear gradient at a flow rate of 0.3 ml/min. The eluents for the positive polarity mode were eluent A (0.1% FA in water) and eluent B (methanol). The eluents for the negative polarity mode were eluent A (5 mM ammonium acetate, pH 9.0) and eluent B (methanol). The solvent gradient was established as follows: 2% B, 1.5 min; 2–100% B, 12 min; 100% B, 14 min; 100–2% B, 14.1 min; 2% B, 16 min. Q ExactiveTM HF-X mass spectrometer was operated in positive/negative polarity mode with spray voltage of 3.2 kV, capillary temperature of 320 ℃, sheath gas flow rate of 35 arb and aux gas flow rate of 10 arb.

### Data analysis

The raw data files generated by UHPLC-MS/MS were processed using the Compound Discoverer 3.0 (CD3.0, Thermo Fisher) to perform peak alignment, peak picking and quantitation for each metabolite. The important parameters were set as follows: retention time tolerance, 0.2 min; actual mass tolerance, 5 ppm; signal intensity tolerance, 30%; signal/noise ratio, 3; minimum intensity, 100,000. Peak intensities were normalized to the total spectral intensity. The normalized data were used to predict the molecular formula based on additive ions, molecular ion peaks and fragment ions. Then, peaks were matched with the mzCloud (https://www.mzcloud.org/) and ChemSpider (http://www.chemspider.com/) database to obtain accurate qualitative and relative quantitative results.

Multivariate statistical analyses including principal component analysis (PCA) and partial least-squares discriminant analysis (PLS-DA) were performed. PLS-DA models were validated with sevenfold cross-validation. Meanwhile, permutation tests were performed to ensure that the PLS-DA models were not overfitting the data. The differential metabolites were selected when the statistically significant threshold of variable importance in the projection (VIP) values obtained from the PLS-DA model were > 1.0 [19]. Statistically significant differences were analyzed using Student’s *t*-test, and *P* < 0.05 was deemed as statistically significant. Urine metabolites passing the VIP threshold (VIP > 1) and fold change  ≥ 2 or  ≤ 0.5 were considered significantly different between two groups [20].

To determine the relevant pathways involved in *T. gondii* infection, altered metabolites were submitted for pathway analysis in webtool MetaboAnalyst 5.0 software (https://www.metaboanalyst.ca) [21]. The MS data have been deposited to the MetaboLights [22] with the data set identifier MTBLS4776 (https://www.ebi.ac.uk/metabolights/MTBLS4776).

## Results

### Altered urine metabolomic profiles during *T. gondii* infection

**Fig. 1:**
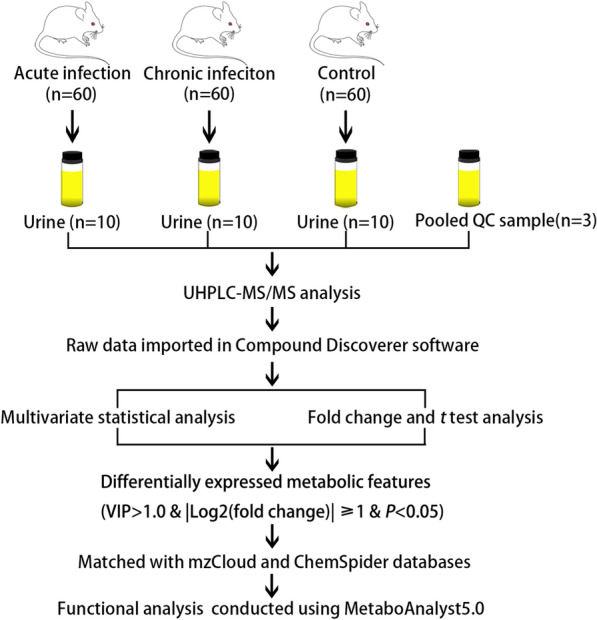
Experimental workflow for the metabolomic study of mouse urine in toxoplasmosis. Urine samples from acute infection group, chronic infection group and healthy control group were centrifuged and analyzed by UHPLC-MS/MS in positive and negative ionization modes. Each group includes ten biological replicates. Quality control (QC) samples were prepared by pooling equal volumes of urine extracts and injected in between blocks of ten samples to ensure analytical reliability and reproducibility

**Fig. 2:**
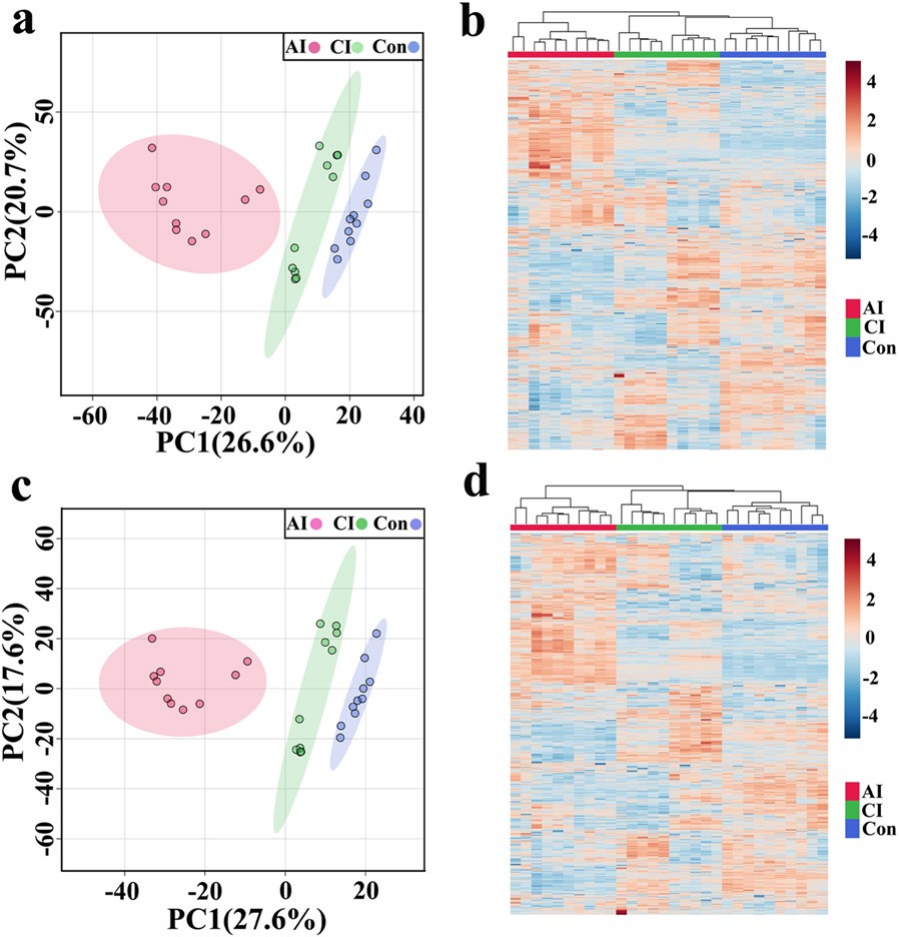
Overall urine metabolic profile during *T. gondii* infection. Principal component analysis (PCA) score scatter plots of identified metabolites obtained from UHPLC-MS/MS fingerprints in ESI + **a** or ESI − mode **c**. Unsupervised hierarchical clustering of metabolic profiling data in ESI + **b** or ESI − mode **d**. Metabolite intensity is normalized so that blue represents low intensity, and reddish brown represents high intensity. Columns were hierarchically clustered based on an average linkage using Pearson correlation coefficients as the distance measure. Sample groups including acutely infected, chronically infected, and healthy controls are labeled as AI, CI and Con, respectively

To investigate the urine metabolic profiles throughout the infection progression, urine extracts from *T. gondii*-infected mice and healthy control mice were analyzed using an UHPLC-MS/MS method. The overall workflow for the sample collection followed by mass spectrometry analysis used in the current study is outlined in Fig. 1. In the positive electrospray ionization (ESI +) mode, 2065 metabolites were putatively identified, whereas 1409 metabolites were detected in the ESI − mode (Additional file 1: Table S1 and Additional file 2: Table S2). As shown in Additional file 3: Fig. S1, QC samples in ESI + and ESI − modes were clustered together tightly in the PCA score plots, indicating satisfactory stability and repeatability of the MS platform. Changes in urine metabolic profiles during *T. gondii* infection were analyzed using two-dimensional PCA score plots. As shown in Fig. 2, no overlap was found among acutely infected, chronically infected and control mice in both ESI + mode and ESI − mode. Heatmaps were constructed based on all identified metabolite features and showed good discrimination between acutely infected and chronically infected or control mice in both ESI + and ESI − modes, which reflects the close relationship between urine metabolic profile and diverse pathological and physiological conditions.

### Patterns of differentially abundant metabolites

**Fig. 3:**
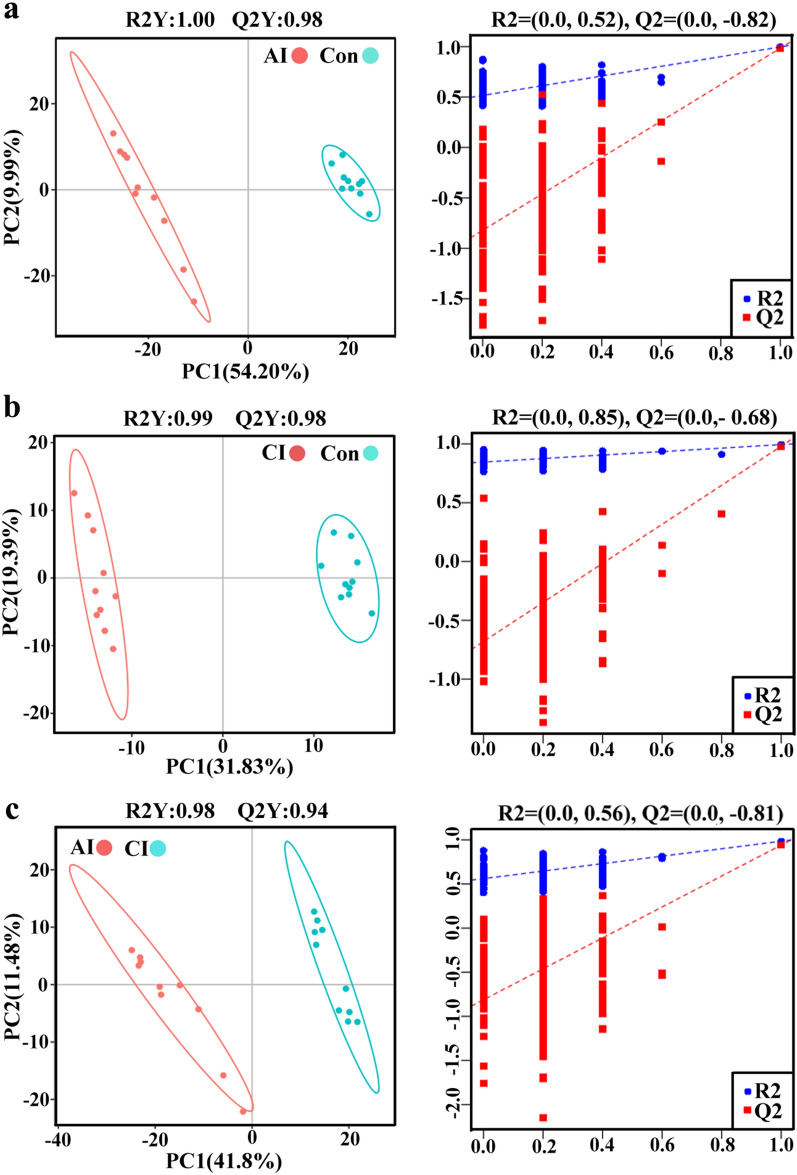
Partial least squares-discriminate analysis (PLS-DA) score plots of metabolic profiling data in ESI + mode with their respective permutation plots for all comparison groups: **a** acutely infected vs. control, **b** chronically infected vs. control and **c** acutely infected vs. chronically infected. Sample groups including acutely infected, chronically infected and healthy control are labeled as AI, CI and Con, respectively

As shown in Fig. 3, the PLS-DA models of acutely infected vs. control (R2 = 1.00, Q2 = 0.98), chronically infected vs. control (R2 = 0.99, Q2 = 0.98) and acutely infected vs. chronically infected group (R2 = 0.98, Q2 = 0.94) showed good discriminations in ESI + mode. Meanwhile, the PLS-DA models of acutely infected vs. control (R2 = 1.00, Q2 = 0.99), chronically infected vs. control (R2 = 0.99, Q2 = 0.98) and acutely infected vs. chronically infected (R2 = 0.99, Q2 = 0.91) also showed good separations in ESI − mode (Additional file 4: Fig. S2). Furthermore, random permutation tests for the PLS-DA models were performed, and the validated models showed no overfitting, supporting that the established PLS-DA model was reliable (Fig. 3 and Additional file 4: Fig. S2).

**Fig. 4:**
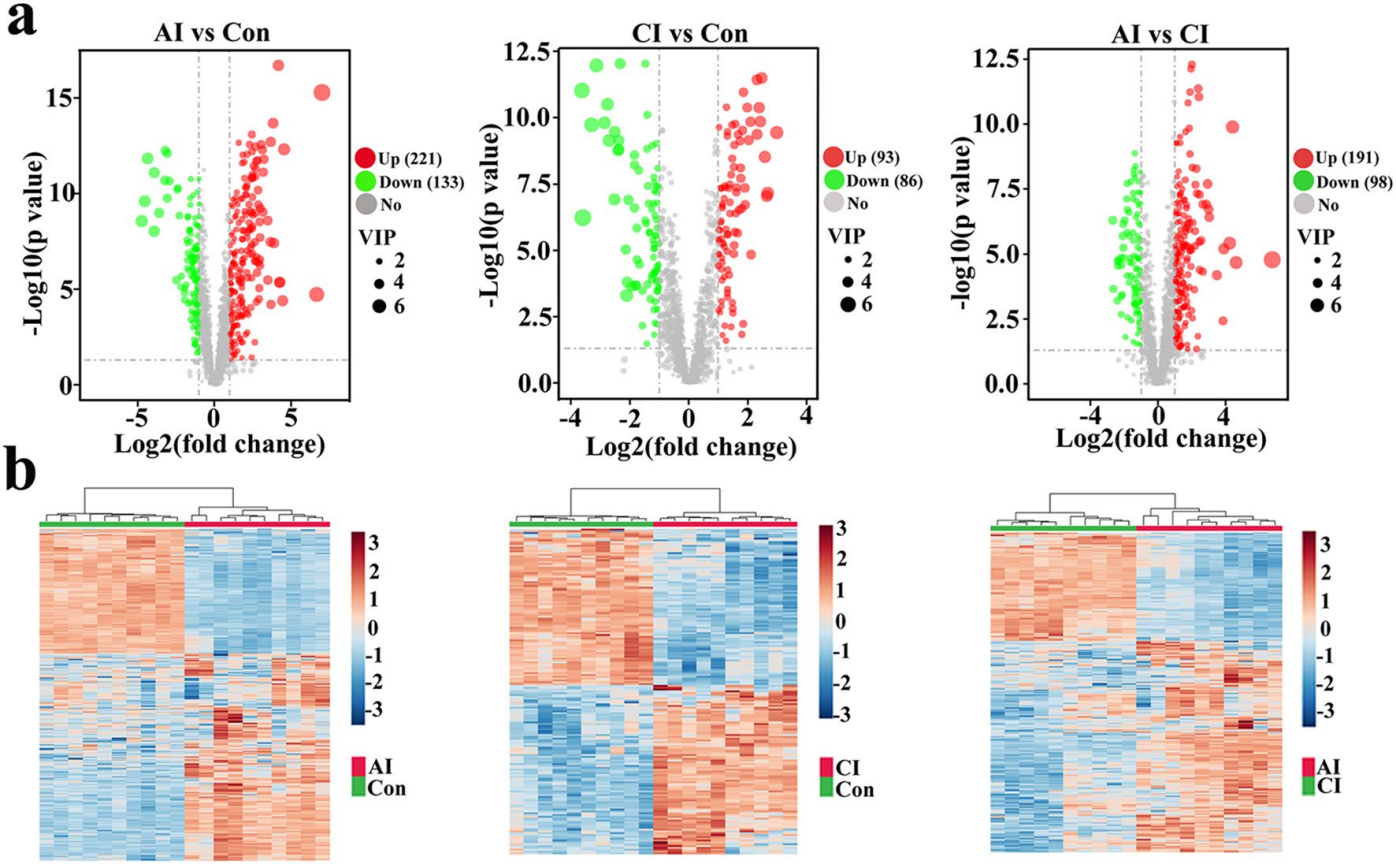
Quantitative analysis of mouse urinary metabolites in response to *T. gondii* infection in ESI + mode. **a** Volcano plot of quantified metabolites in acutely infected vs. control group, chronically infected vs. control group and acutely infected vs. chronically infected group. In this plot, the *x*-axis is log2 fold-change, which shows the direction of the change (negative scale is decrease and positive scale is increase) in the levels of metabolite intensity, while the *y*-axis is the -log10 *P*-value, which shows the significance of the change. **b** Comparison of significantly changed metabolites between acutely infected, chronically infected and control mice in ESI + mode. Each row represents data for a particular metabolite, and each column represents a urine sample. The colors reddish brown and blue reflect increasing and reduced metabolite levels, respectively. Columns are hierarchically clustered based on an average linkage using Pearson correlation coefficients as the distance measure. Sample groups including acutely infected, chronically infected and healthy control are labeled as AI, CI and Con, respectively

Metabolites with VIP value > 1.0, *P* value < 0.05 and |log2 fold change| $\ge$1 were the potential differential metabolites. As shown in Fig. 4a, compared to the control group, 354 differential metabolites in the acute infection group were detected in ESI + mode, among which the levels of 221 metabolites were elevated, while 133 metabolites were downregulated. Meantime, in the chronic infection group in ESI + mode, we identified 93 metabolites with elevated levels and 86 metabolites with decreased levels. In the ESI − mode, 239 differential metabolites were identified in the comparison between the acutely infected and normal group, whereas 145 differential metabolites were detected between chronically infected and control mice (Additional file 5: Fig. S3a). As shown in Fig. 4b and Additional file 5: Fig. S3b, heatmaps constructed based on differential metabolites exhibit excellent separations.

**Fig. 5:**
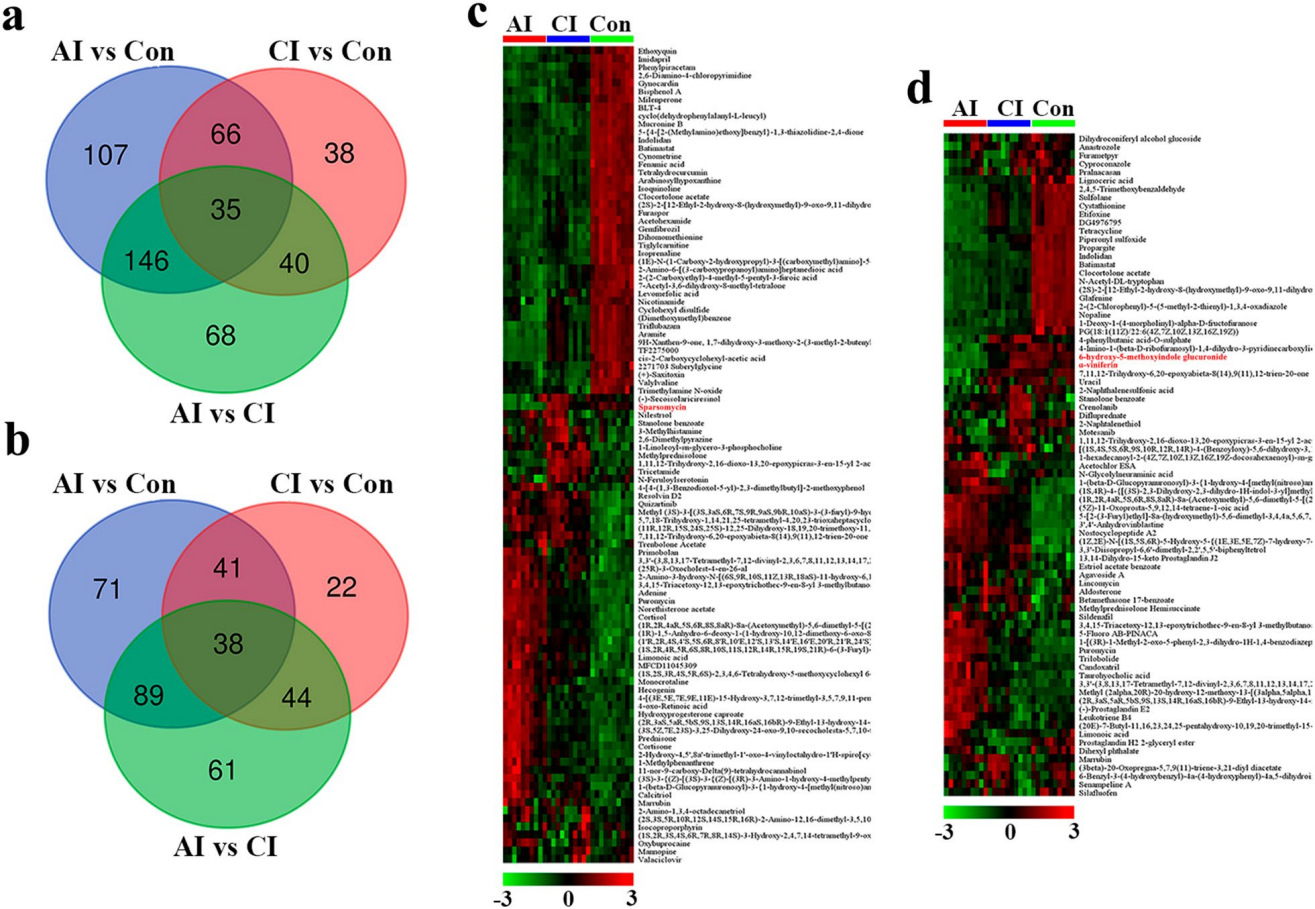
Comparison of the mouse urine metabolomes during *T. gondii* infection. Venn diagram representations of differential metabolites across comparison groups showing unique and common metabolites in **A** ESI + mode and **B** ESI – mode. Heatmaps displaying the common differential metabolites detected between acutely infected vs. control and chronically infected vs. control in ESI + mode **c** and ESI − mode **d**, respectively. Columns were hierarchically clustered based on an average linkage using Pearson correlation coefficients as the distance measure. Green denotes low metabolite intensity, whereas red shows high metabolite intensity

Of the 500 differential metabolites detected in ESI + mode, 35 were common across the three comparison pairs (Fig. 5a). Approximately one fifth of the differential metabolites were common between AI vs. Con and CI vs. Con, which is shown in Fig. 5c. Meanwhile, in the ESI − mode, there were 366 differential metabolites, of which 38 were common across the three comparison pairs (Fig. 5b). A total of 79 differential metabolites were common between AI vs. Con and CI vs. Con, which is shown in Fig. 5d.

### Altered metabolic pathways during the infection course

**Fig. 6:**
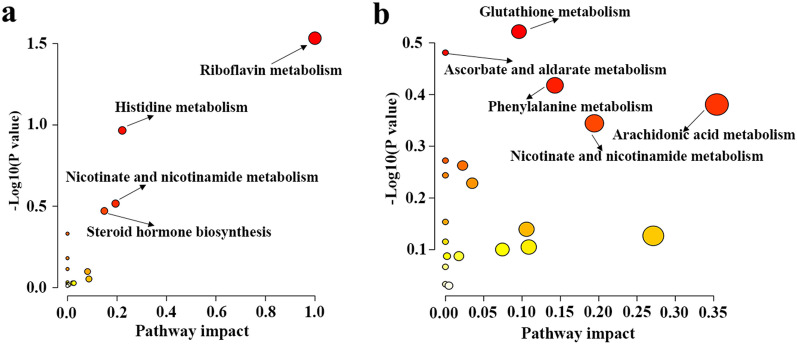
Highly influenced pathways during *T. gondii* infection. Small *P* values and high pathway impact factors indicate that the pathway is greatly influenced. **a** Metabolic pathways were perturbed at the acute infection stage. **b** Metabolic pathways were perturbed at the chronic infection stage

Pathway analysis was performed to determine the significantly affected pathways during *T. gondii* infection. Sixteen pathways were disturbed in the acute infection stage. As shown in Fig. 6a, four metabolic pathways were shown to be remarkably affected, which include pathways related to the metabolism of riboflavin metabolism, histidine metabolism, nicotinate and nicotinamide metabolism, and steroid hormone biosynthesis. At the chronic infection stage, 20 pathways were affected by *T. gondii* infection. Metabolic pathways such as arachidonic acid metabolism, nicotinate and nicotinamide metabolism, phenylalanine metabolism, glutathione metabolism, ascorbate and aldarate metabolism, were considerably disrupted (Fig. 6b).

## Discussion

Quantitative metabolomic profiling has recently emerged as a powerful approach for surveillance and investigation of health-related states, which is now widely applied in cancer and infectious diseases [23–25]. The previous omics reports on toxoplasmosis and other protozoal diseases mainly focused on changes of biochemical compounds in serum, but seldom in urine, which might result from large inter- and intraindividual variations [26]. The present work fills this lacuna and demonstrates the potential of an LC-MS/MS-based metabolomics approach for identifying *T. gondii*-infected mice from healthy control subjects based on their characteristic urine metabolite profiles. Here, we detected a wide range of differential metabolic features at acute and chronic infection stages. Remarkable metabolic disturbances were found in multiple metabolic pathways, including amino acid metabolism, fatty acid metabolism, and nicotinate and nicotinamide metabolism. To the best of our knowledge, this is the first study on urine metabolic profiling for toxoplasmosis.

In our previous study, we carried out serum metabolomics analysis to unravel the metabolic signatures perturbed by *T. gondii* infection in mouse [27]. PCA scores plots derived from non-targeted metabolite profiling of serum failed to distinguish chronically infected mice from control group in both ion modes. However, PCA plots based on the urine data in this study showed clear separation among control, chronically infected and acutely infected mice, which indicated urinary metabolites are more sensitive to alterations caused by *T. gondii* infection. In both serum and urine, a dramatic metabolic disturbance occurred at acute infection stages, which might be caused by multiorgan dysfunctions triggered by rapid proliferation of parasites [28]. Later, the immune system reacts to pathogens and restores homeostasis, which results in fewer differential metabolites. It is noticeable that, compared to serum, there were seven times as many differential metabolites in urine at chronic infection stage, which revealed that fluctuations in the levels of endogenous metabolites in urine better reflect the pathophysiological conditions during toxoplasmosis progression.

Compared with the control group, 101 and 79 common differentials were detected between AI vs. Con and CI vs. Con in ESI + and ESI − mode, respectively (Fig. 5c and d). Interestingly, all the above differential metabolites showed the same variation tendency during parasite infection, except sparsomycin (ESI +) 6-hydroxy-5-methoxyindole glucuronide (ESI −) and α-viniferin (ESI −). Sparsomycin is not a naturally occurring metabolite, which has been found in blood of those individuals exposed to this compound [29]. However, the identification and biological function of sparsomycin in toxoplasmosis need further verification and investigation. 6-Hydroxy-5-methoxyindole glucuronide is a natural animal and human metabolite and is generated in the liver by UDP glucuonyltransferase, which is found significantly downregulated in urine samples from Alzheimer’s disease mouse model [30]. In another study, the level of 6-hydroxy-5-methoxyindole glucuronide is elevated in a diabetic kidney disease (DKD) mouse model, which indicates this metabolite might be involved in DKD development [31]. α-Viniferin is a food-derived metabolite. More evidence has shown that α-viniferin displayed a broad range of pharmacological activities, including anti-Alzheimer’s disease and anti-inflammatory [32, 33]. However, its biological function in toxoplasmosis needs further exploration. As shown in Fig. 5, adenine was common across the three comparison pairs in ESI + mode, and its abundance is positively associated with the disease severity. Adenine is a purine nucleobase, and its final metabolite is uric acid. Previous studies showed that high level of blood adenine is nephrotoxic, and abnormal plasma adenine concentration is involved in chronic kidney failure [34, 35]. Histopathological changes in mouse kidney infected with *T. gondii* pru strain indicated that acute nephritis occurs in acute infection mouse [18], which might result in the adenine perturbation. Leukotriene B4 is a potent chemoattractant for neutrophils and is involved in progressive glomerular damage [36], which is found significantly upregulated in infection groups. Meanwhile, taurohyocholic acid was found significantly increased in the urine of *T. gondii*-infected mice. Taurohyocholic acid is found predominantly in the bile of mammals. Several urinary metabolomic studies found taurohyocholic acid was markedly elevated in hepatitis B cirrhosis patients [37, 38]. The roles of taurohyocholic acid in liver disease caused by *Toxoplasma* infection need further elucidation.

Metabolomics research on urine has recently emerged as a powerful technique to study the associations between parasite infections and health effects. For example, levels of kynurenic acid (KA) and quinolinic acid (QA) were found to be elevated in urine in male mice infected by *Plasmodium berghei* ANKA [39]. There is evidence that KA and QA are involved in the pathogenesis of cerebral malaria [40]. Intriguingly, KA and QA were upregulated in the acute infection stage in the present study, which might be related to the central nervous system injury caused by *T. gondii* invasion. Hu et al. found that xanthurenic acid was decreased, whereas naphthalenesulfonic acid was increased following *Schistosoma japonicum* infection. Additionally, both compounds showed the most discriminatory power and had the potential to be the most powerful targets for the early diagnosis of schistosomiasis [41]. Notably, xanthurenic and naphthalenesulfonic acids were significantly altered during *T. gondii* infection in the present study, which indicates that further validations of those potential biomarkers in large sample sizes are necessary.

## Conclusions

Our urinary metabolomics workflow is relatively straightforward, and we have demonstrated that urine has the potential to inform about variation status in protozoal diseases. The metabolite fingerprint revealed comprehensive metabolic abnormalities in acute toxoplasmosis and chronic infection. These differential metabolites identified in this study are useful to understand the pathogenic mechanism of *T. gondii*, and they are also valuable for the clinical noninvasive diagnosis or therapy of toxoplasmosis.

## Supplementary Information

**Additional file 1: Table S1.** The putative metabolites identified in ESI+ mode.

**Additional file 2: Table S2.** The putative metabolites identified in ESI- mode.

**Additional file 3: Figure. S1.** Principal components analysis (PCA) scores plot generated from the detected metabolic features across all urine samples including QC samples in the positive (a) and negative (b) ion modes, respectively. Red dots represent urine samples, and green dots represent QC samples.

**Additional file 4: Figure. S2.** Partial least squares-discriminate analysis (PLS-DA) score plots of metabolic profiling data in ESI- mode with their respective permutation plots for all comparison groups: (a) acutely infected vs. control, (b) chronically infected vs. control, and (c) acutely infected vs. chronically infected. Sample groups including acutely infected, chronically infected, and healthy control are labeled as AI, CI and Con, respectively.

**Additional file 5: Figure. S3.** Quantitative analysis of mouse urinary metabolites in response to *T. gondii *infection in ESI- mode. (a) Volcano plot of quantified metabolites in acutely infected vs. control group, chronically infected vs. control group, and acutely infected vs. chronically infected group. In this plot, the x-axis is log 2 fold-change, which shows the direction of the change (negative scale is decrease and positive scale is increase) in the levels of metabolite intensity, while the y-axis is the -log10 *P*-value, which shows the significance of the change. (b) Comparison of significantly changed metabolites between acutely infected, chronically infected, and control mice in ESI+ mode. Each row represents data for a particular metabolite, and each column represents a urine sample. The colors reddish brown and blue reflect increasing and reduced metabolite levels, respectively. Columns were hierarchically clustered based on an average linkage using Pearson correlation coefficients as the distance measure. Sample groups including acutely infected, chronically infected, and healthy control are labeled as AI, CI and Con, respectively.

## Data Availability

The mass spectrometry proteomics data have been deposited to the MetaboLights [22] with the data set identifier MTBLS4776 (https://www.ebi.ac.uk/metabolights/MTBLS4776).

## Abbreviations

AI: Acute infection
CI: Chronic infection
Con: Control
DKD: Diabetic kidney disease
KA: Kynurenic acid
PCA: Principal component analysis
PLS-DA: Partial least squares discriminant analysis
QA: Quinolinic acid
QC: Quality control
VIP: Variable importance in the projection
