# Supplementary figures and images for: Investigation of urine metabolome of BALB/c mouse infected with an avirulent strain of Toxoplasma gondii
Source: Parasit Vectors. 2022 Jul 29;15:271. doi: 10.1186/s13071-022-05408-2 (PMC9338554; doi:10.1186/s13071-022-05408-2)

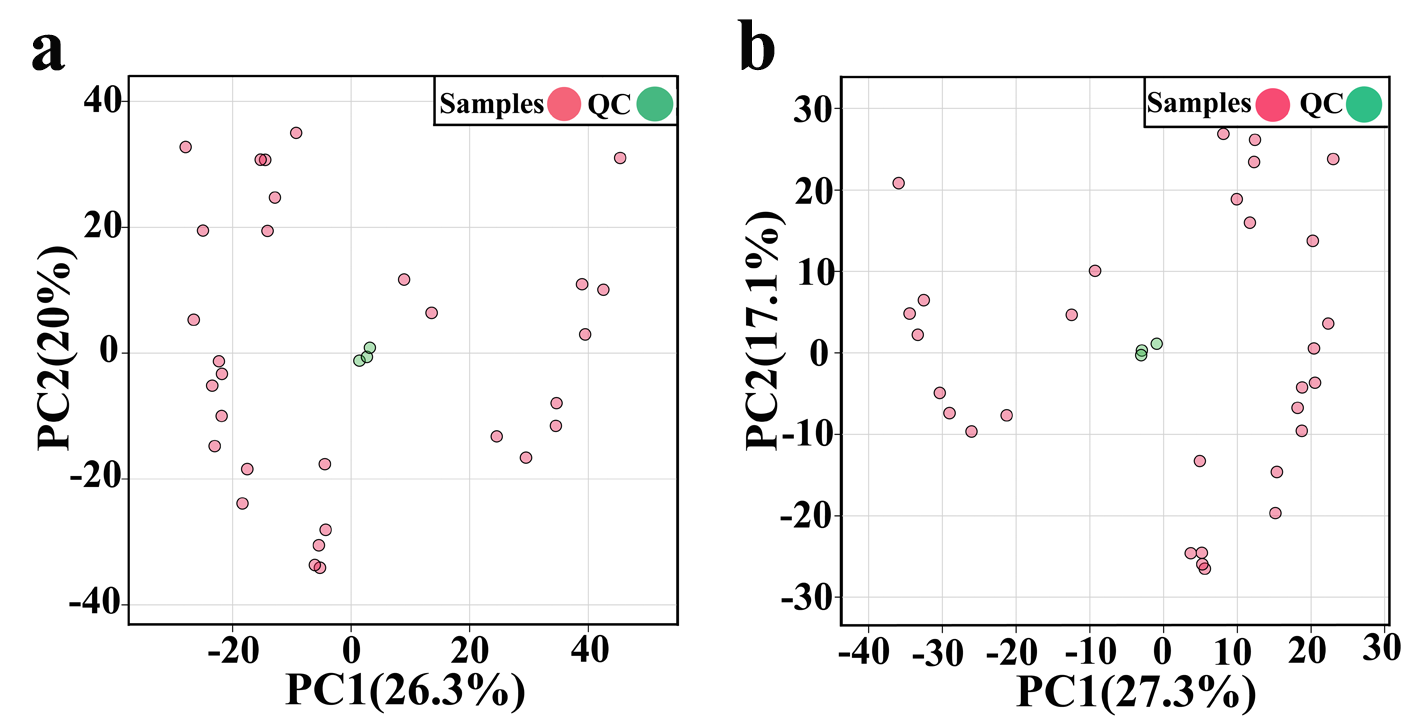

Supplement: Supplementary file 3 — Additional file 3: Figure. S1. Principal components analysis (PCA) scores plot generated from the detected metabolic features across all urine samples including QC samples in the positive (a) and negative (b) ion modes, respectively. Red dots represent urine samples, and green dots represent QC samples. [file 13071_2022_5408_MOESM3_ESM.tif]

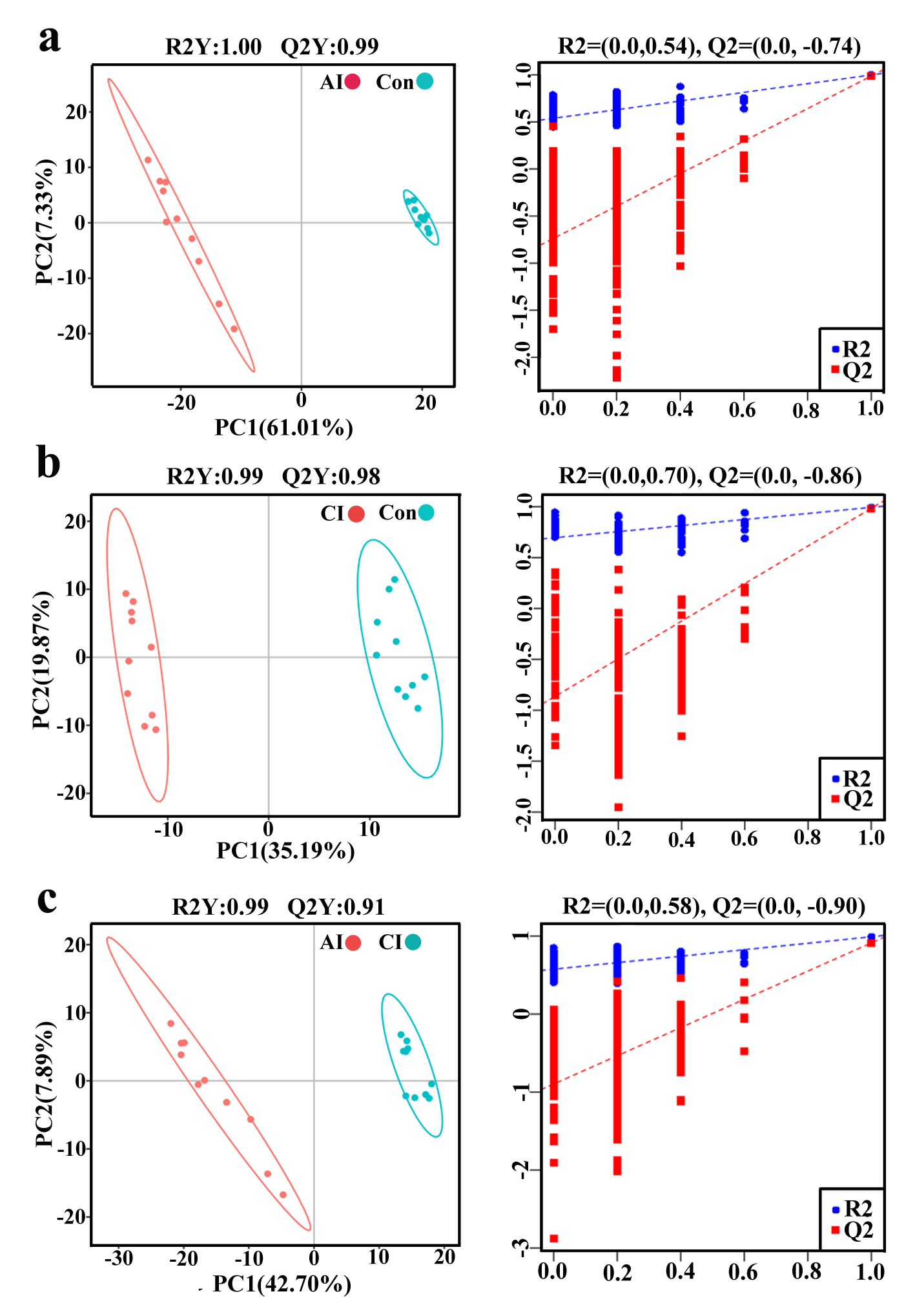

Supplement: Supplementary file 4 — Additional file 4: Figure. S2. Partial least squares-discriminate analysis (PLS-DA) score plots of metabolic profiling data in ESI- mode with their respective permutation plots for all comparison groups: (a) acutely infected vs. control, (b) chronically infected vs. control, and (c) acutely infected vs. chronically infected. Sample groups including acutely infected, chronically infected, and healthy control are labeled as AI, CI and Con, respectively. [file 13071_2022_5408_MOESM4_ESM.tif]

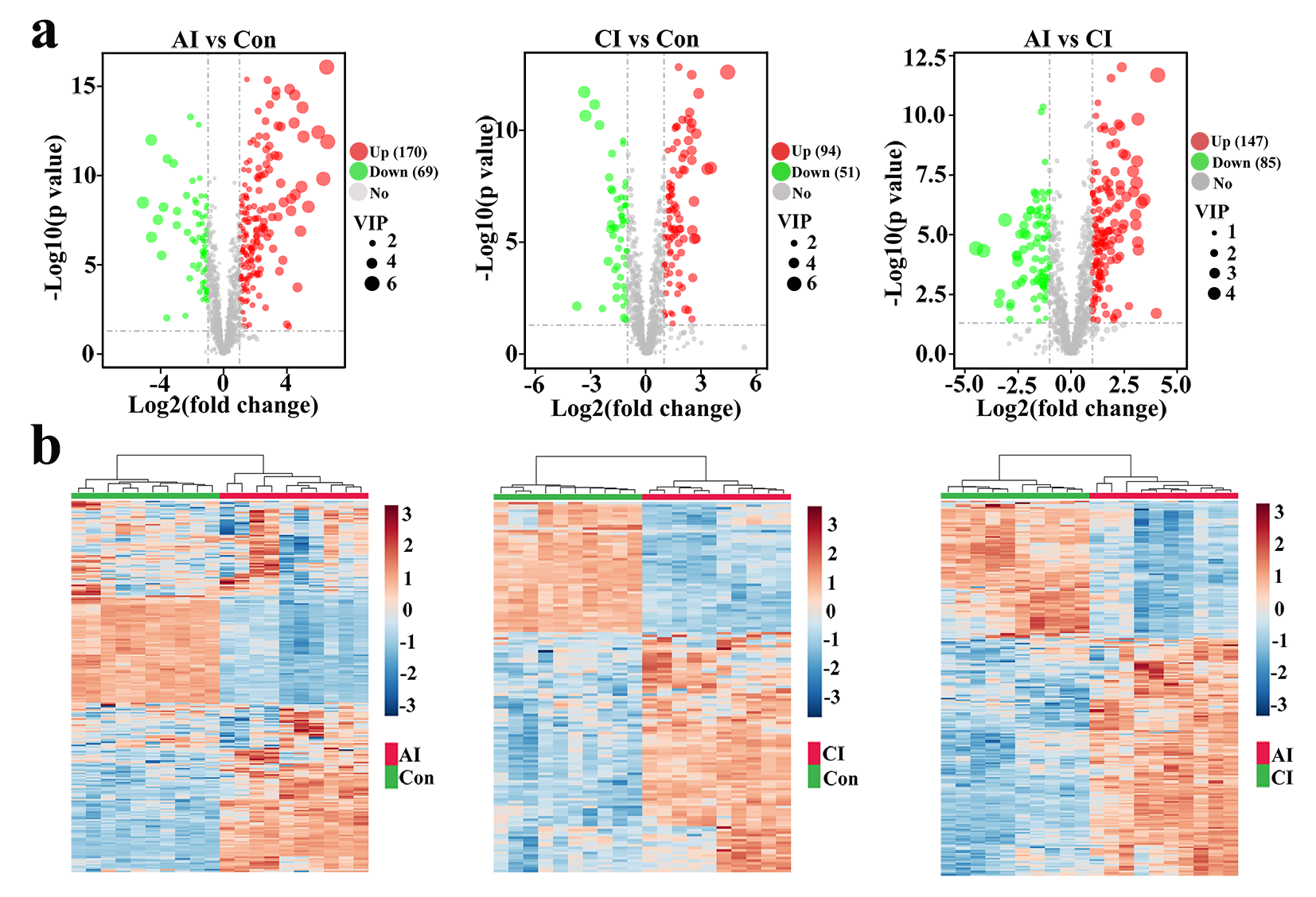

Supplement: Supplementary file 5 — Additional file 5: Figure. S3. Quantitative analysis of mouse urinary metabolites in response to T. gondii infection in ESI- mode. (a) Volcano plot of quantified metabolites in acutely infected vs. control group, chronically infected vs. control group, and acutely infected vs. chronically infected group. In this plot, the x-axis is log 2 fold-change, which shows the direction of the change (negative scale is decrease and positive scale is increase) in the levels of metabolite intensity, while the y-axis is the -log10 P-value, which shows the significance of the change. (b) Comparison of significantly changed metabolites between acutely infected, chronically infected, and control mice in ESI+ mode. Each row represents data for a particular metabolite, and each column represents a urine sample. The colors reddish brown and blue reflect increasing and reduced metabolite levels, respectively. Columns were hierarchically clustered based on an average linkage using Pearson correlation coefficients as the distance measure. Sample groups including acutely infected, chronically infected, and healthy control are labeled as AI, CI and Con, respectively. [file 13071_2022_5408_MOESM5_ESM.tif]
